# Supplementary material for: A temporally dynamic Foxp3 autoregulatory transcriptional circuit controls the effector Treg programme
Source: EMBO J. 2018 Jul 10;37(16):e99013. doi: 10.15252/embj.201899013 (PMC6092677; doi:10.15252/embj.201899013)
Supplement: Supplementary file 1 — Expanded View Figures PDF [file EMBJ-37-e99013-s001.pdf]

Expanded View Figures

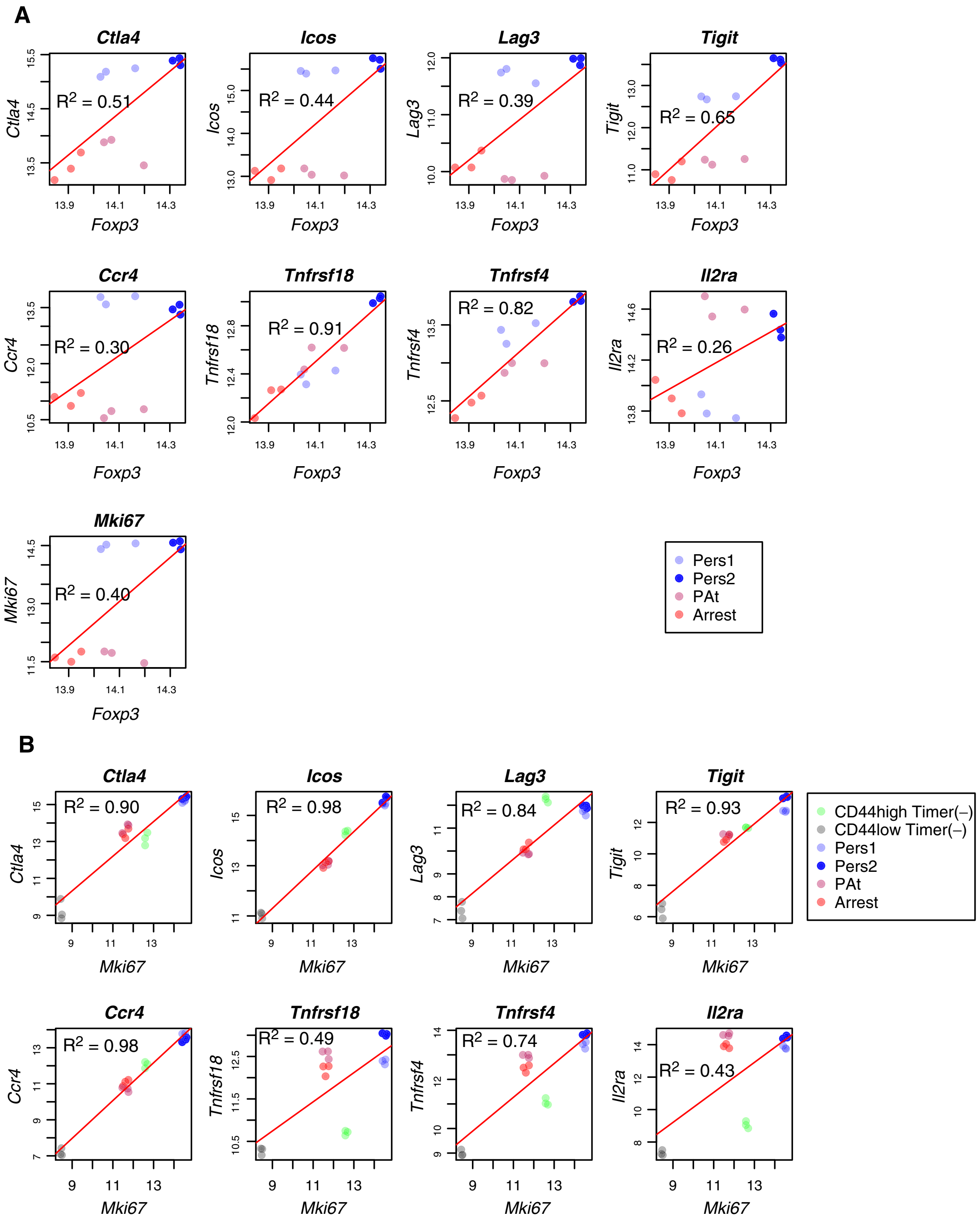

Figure EV1. Analysis of correlations between *Foxp3* and *Mki67* in RNA-seq data from Fig 5.

A, B Scatter plots showing the correlations between the indicated transcripts (normalised log2 of read counts) and *Foxp3* transcripts and *Mki67* transcripts. Note that Foxp3$^+$ cells only are shown in (A). Red lines indicate linear regression line, and R-square values are shown. Group designations are shown by colours (see legend), *n* = 3.

© 2018 The Authors

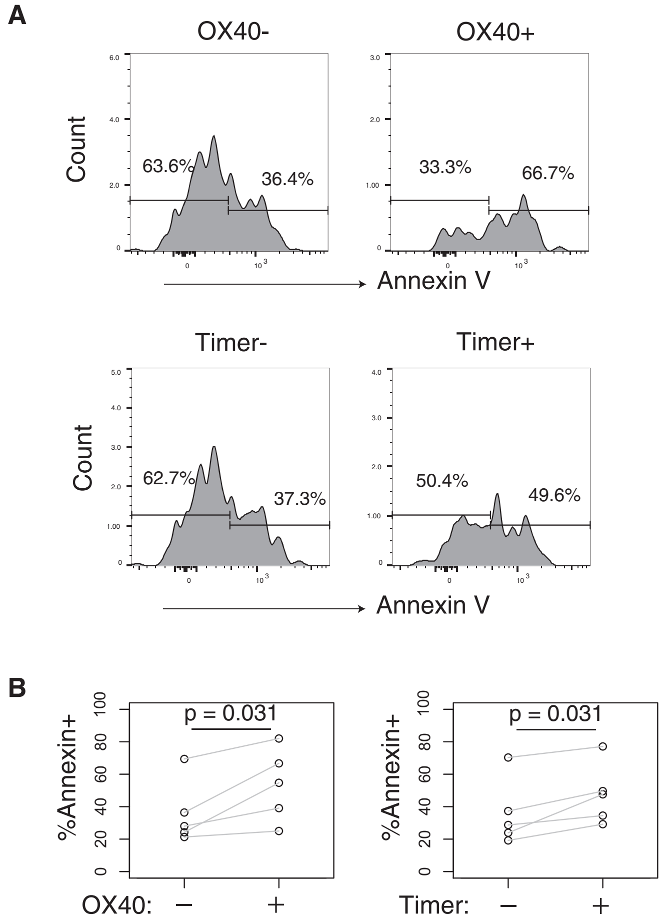

Figure EV2. OX40$^+$ and Timer$^+$ T cells are enriched with pre-apoptotic cells.

A *Foxp3*-Tocky mice were sensitised by application of 3% oxazolone to abdominal skin. 5 days later, both ears were challenged with 1% oxazolone. 96 h later, ears were removed and CD4$^+$ T cells stained for Annexin V. Shown are histograms, gated on viable CD4$^+$ T-cells, displaying Annexin V fluorescence in OX40$^-$ or OX40$^+$ (Top panel) or Timer$^-$ or Timer$^+$ *Foxp3*-Tocky CD4$^+$ T cells (bottom panel).

B Summary data of Annexin V$^+$ cells within paired samples of OX40$^-$ and OX40$^+$ (left) or Timer$^-$ and Timer$^+$ (right) CD4$^+$ skin-infiltrating T cells (n = 5). Data are combined from two independent experiments. Statistical test by Mann–Whitney *U*-test.

 © 2018 The Authors
